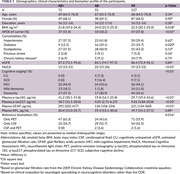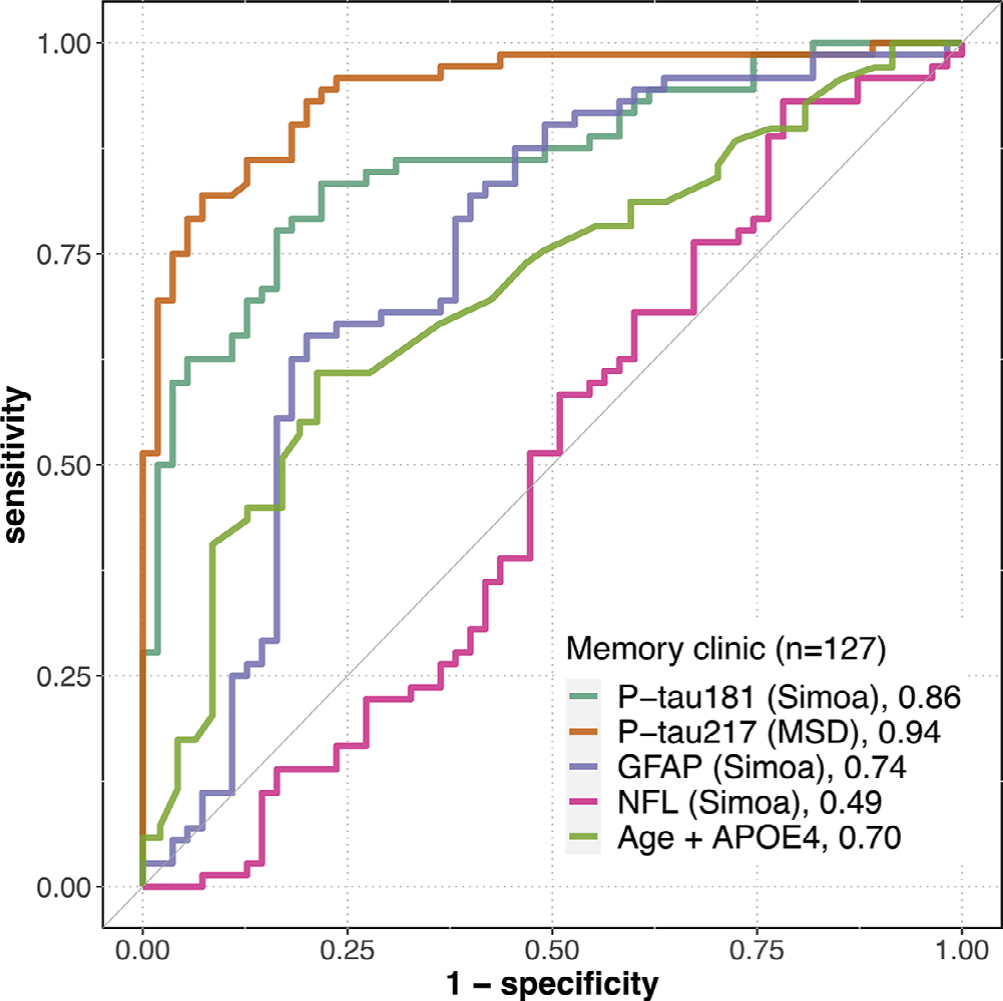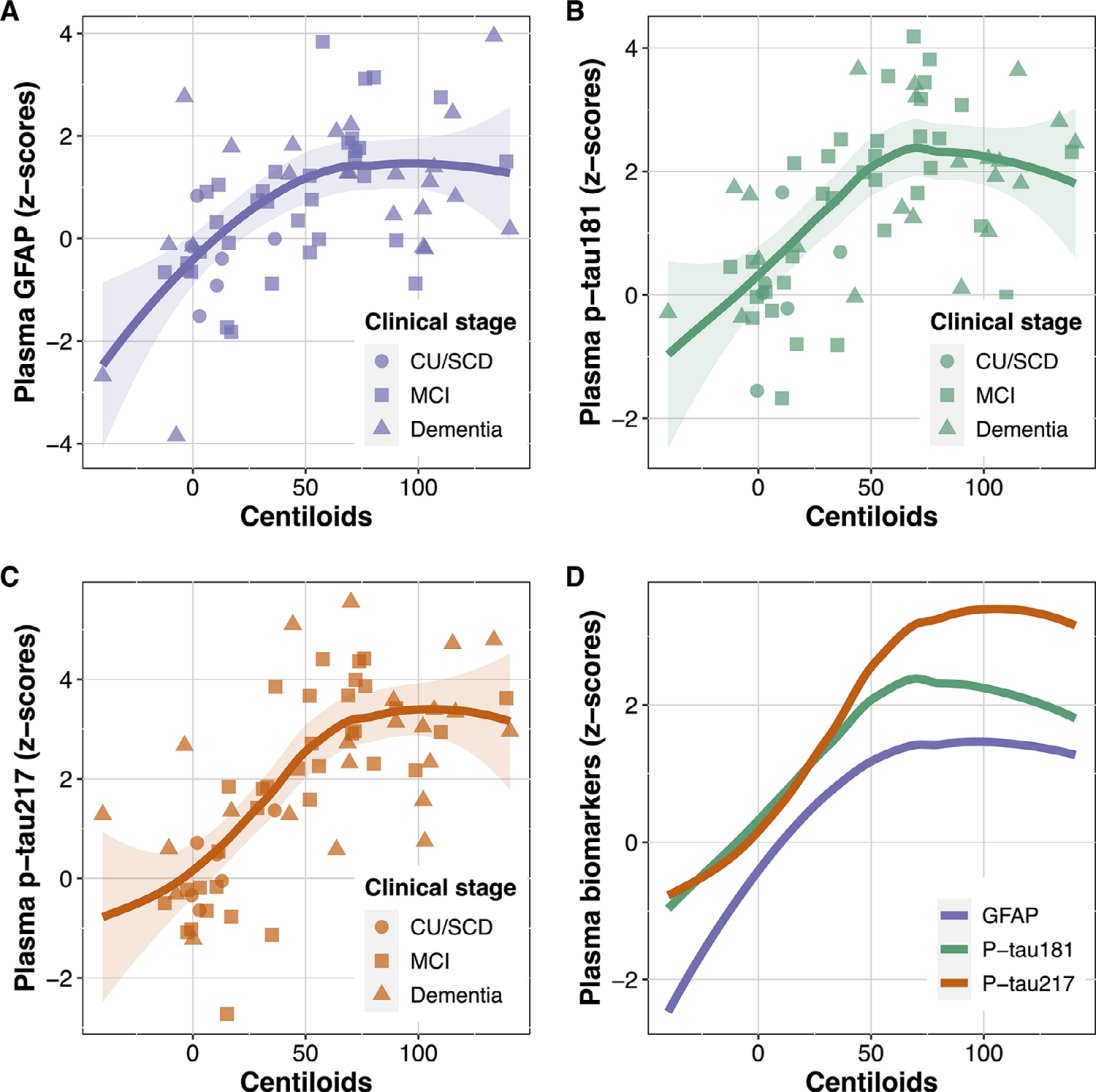# Evaluation of Plasma Glial Fibrillary Acidic Protein in Real‐life Memory Clinics in Thailand

**DOI:** 10.1002/alz.091107

**Published:** 2025-01-09

**Authors:** Thanapoom Taweephol, Thanakit Pongpitakmetha, Poosanu Thanapornsangsuth, Kittithatch Booncharoen, Jedsada Khieukhajee, Watayuth Luechaipanit, Thanaporn Haethaisong, Adipa Chongsuksantikul, Yuttachai Likitjaroen, Thiravat Hemachudha

**Affiliations:** ^1^ Faculty of Medicine, Chulalongkorn University, Bangkok Thailand; ^2^ Chula Neuroscience Center, King Chulalongkorn Memorial Hospital, Bangkok Thailand; ^3^ Memory Clinic, King Chulalongkorn Memorial Hospital, Bangkok Thailand; ^4^ Thai Red Cross Emerging Infectious Diseases Health Science Centre, King Chulalongkorn Memorial Hospital, Bangkok Thailand; ^5^ Neurology Center, Phyathai 1 Hospital, Bangkok Thailand; ^6^ Neurocognitive Unit, Division of Neurology, Faculty of Medicine, Chulalongkorn University, Bangkok Thailand; ^7^ Neurological Institute of Thailand, Ratchathewi, Bangkok Thailand; ^8^ Division of Neurology, Department of Medicine, Faculty of Medicine, Chulalongkorn University, Bangkok Thailand

## Abstract

**Background:**

The roles of reactive astrogliosis in response to the accumulation of amyloid‐β (Aβ) and early tau phosphorylation in Alzheimer’s disease (AD) have been more elucidated. Plasma glial fibrillary acidic protein (GFAP) has taken the spotlight, following phosphorylated tau (p‐tau), in evaluating patients with AD. In this study, we aimed to assess the performance of plasma GFAP compared to other biomarkers.

**Method:**

We consecutively evaluated patients with cognitive complaints from two memory clinics in Bangkok, Thailand: Memory Clinic, King Chulalongkorn Memorial Hospital and the Neurology Clinic at the Prasat Neurological Institute. Individuals who underwent CSF analysis for core AD biomarkers or Amyloid PET to predict AD status were classified as Aβ positive (Aβ+) or negative (Aβ‐). The plasma biomarkers were measured, and their diagnostic performances were assessed using receiver operating characteristic curve. Cohen’s d was utilized to estimate the effect size of each plasma biomarkers between the Aβ+ and Aβ‐ groups. Loess regression models were employed to illustrate the trends between plasma biomarker levels and amyloid PET centiloid.

**Result:**

Among 127 participants, 87 were female. The median age was 68 years (interquartile range: 61, 75). (Table 1) There were 72 patients with Aβ+. Plasma GFAP demonstrated an area under the curve (AUC) of 0.74 (95% confidence interval [CI] 0.65‐0.84) for detecting Aβ+ in patients with cognitive complaints. Comparatively, plasma p‐tau217, p‐tau181, neurofilament light chain, and the base model (BM, comprising age and *APOE* status) had AUC of 0.94 (95%CI 0.90‐0.98), 0.86 (95%CI 0.80‐0.92), 0.49 (95%CI 0.39‐0.60), and 0.70 (95%CI 0.61‐0.80), respectively. Unlike plasma p‐tau, the AUC of plasma GFAP showed no significant difference from the BM (p=0.555). (Figure 1) Plasma levels of GFAP, p‐tau217, and p‐tau181 have large effects on determining Aβ status, with Cohen’s d = 0.81 (95%CI 0.44‐1.18), 2.23 (95%CI 1.78‐2.68), and 1.54 (95%CI 1.14‐1.95), respectively. Furthermore, the loess regression showed an early increase in plasma GFAP, together with p‐tau in memory clinic patients (n=63). (Figure 2)

**Conclusion:**

Plasma GFAP increases alongside p‐tau from early stages of AD, as indicated by amyloid PET centiloid. Nevertheless, the diagnostic performance does not differ from that of the BM.